# Characterization of Transposable Elements in the Ectomycorrhizal Fungus *Laccaria bicolor*

**DOI:** 10.1371/journal.pone.0040197

**Published:** 2012-08-03

**Authors:** Jessy Labbé, Claude Murat, Emmanuelle Morin, Gerald A. Tuskan, François Le Tacon, Francis Martin

**Affiliations:** 1 Unité Mixte de Recherche de l'Institut National de la Recherche Agronomique – Lorraine Université ‘Interactions Arbres/Microorganismes’, Centre de Nancy – Champenoux, France; 2 BioSciences Division, Oak Ridge National Laboratory, Oak Ridge, Tennessee, United States of America; University of California Riverside, United States of America

## Abstract

**Background:**

The publicly available *Laccaria bicolor* genome sequence has provided a considerable genomic resource allowing systematic identification of transposable elements (TEs) in this symbiotic ectomycorrhizal fungus. Using a TE-specific annotation pipeline we have characterized and analyzed TEs in the *L. bicolor* S238N-H82 genome.

**Methodology/Principal Findings:**

TEs occupy 24% of the 60 Mb *L. bicolor* genome and represent 25,787 full-length and partial copy elements distributed within 171 families. The most abundant elements were the *Copia*-like. TEs are not randomly distributed across the genome, but are tightly nested or clustered. The majority of TEs exhibits signs of ancient transposition except some intact copies of terminal inverted repeats (TIRS), long terminal repeats (LTRs) and a large retrotransposon derivative (*LARD*) element. There were three main periods of TE expansion in *L. bicolor*: the first from 57 to 10 Mya, the second from 5 to 1 Mya and the most recent from 0.5 Mya ago until now. LTR retrotransposons are closely related to retrotransposons found in another basidiomycete, *Coprinopsis cinerea*.

**Conclusions:**

This analysis 1) represents an initial characterization of TEs in the *L. bicolor* genome, 2) contributes to improve genome annotation and a greater understanding of the role TEs played in genome organization and evolution and 3) provides a valuable resource for future research on the genome evolution within the *Laccaria* genus.

## Introduction

The basidiomycete *Laccaria bicolor* (Maire) P.D. Orton is an ectomycorrhizal fungus living in symbiosis with various woody host plants. Symbiotic plant-fungus associations play a fundamental role in biology and ecology of forest trees, affecting growth, water and nutrient absorption, and providing protection from root diseases [1]. *L. bicolor* is an early stage ectomycorrhizal symbiont able to establish itself on seedlings, but it also colonizes roots of adult conifer and hardwood trees. *L. bicolor* is characterized by its ubiquitous geographical distribution and lack of strict host specificity. This habit has been associated with its relatively large genome, containing the largest fungal gene repertoire and an abundance of multiple gene families relative to other ectomycorrhizal fungi, which in turn facilitates its adaptation to and interaction with various hosts [2]. The whole-genome sequence (WGS) of *L. bicolor* S238N-H82 provided the first ectomycorrhizal symbiont blueprint, fostering new insights into the nature of the genome of one of the largest groups of fungi [2]. At 60 Mb, the draft genome of *L. bicolor* is larger than that of previously published fungal genomes. The size is partly explained by unprecedented transposon diversity – more types than any other fungi studied to date.

Transposable elements (TEs) are short, mobile, conserved segments of DNA that can replicate and randomly insert copies within genomes. They have had an important influence on the evolution of eukaryote genomes [3]. These elements often constitute a large proportion of eukaryotic genomes, e.g., ∼45% of the human genome [4] and 50 to 80% of some grass genomes [5], and yet alternatively only 9.7% of the fungal *Magnaporthe grisea* genome [6]. Although TEs were first identified in fungi in the yeast *Saccharomyces cerevisiae* [7], conventional genetic studies with *Ascobolus immersus* mutants established their existence in filamentous fungi [8]. *Ty1-Copia* retroelements have been reported in three ectomycorrhizal basidiomycetes, *L. bicolor*, *Pisolithus macrocarpus* and *Pisolithus* MH155 by Diez *et* *al*. [9].

TEs are also known to affect genome organization; recombination between elements at different sites can lead to large–scale chromosomal rearrangements. Some TE insertions may become fixed and play a role in gene function [10], and indeed, TE insertions next to genes can modify gene expression patterns while insertions within genes can negatively impact transcription and gene function. In general, proliferation of TEs induces potentially deleterious effects. To avoid such effects eukaryotes have developed mechanisms that limit TE activity. Paradoxically, TE existence depends on the survival of eukaryote genome. It has been reported that TEs have developed silencing mechanisms directing their integration to specific parts of the genome with minimal damage [11]. Thus, there is no doubt that TEs have evolved in close association with eukaryote genomic DNA.

**Table 1:** Transposable elements characterized in *Laccaria bicolor* genome.

TE type	Family	Copy number	Full length/incomplete	DNA amount (Mb)	TE composition in genome (%)
***Class I***					
LTR	78	6611	110/6501	3.99	6.65
***ERV***	1	7	4/3	0.03	0.05
* Copia*-like	46	3382	57/3325	2.23	3.72
* Gypsy*-like	27	3085	47/3038	1.71	2.85
* LARD*	3	118	1/117	0.05	0.08
* DIRS*	1	19	1/18	0.01	0.01
**Non-LTR**	9	1273	23/1250	2.21	3.68
* Tad*	2	22	17/5	1.6	2.60
* Deceiver*	1	1	1/0	0.02	0.03
* L1*	1	1	0/1	0.01	0.02
* LINEs*	5	1249	5/1244	0.58	0.96
Total Class I	87	7884	133/7751	6.20	10.33
***Class II***					
TIRs	23	6369	692/5677	2.85	4.75
* Helitrons*	1	215	2/213	0.08	0.13
* MITEs*	4	249	103/146	0.06	0.10
Total Class II	28	6833	797/6036	2.99	4.98
TE fragments a	56	11070	–	5.33	8.88
Total TE	175	25787	930/13787	14.52	24.2

a Others elements truncated, unclassified, unfound matches and without category (named no category).

**Figure 1:**
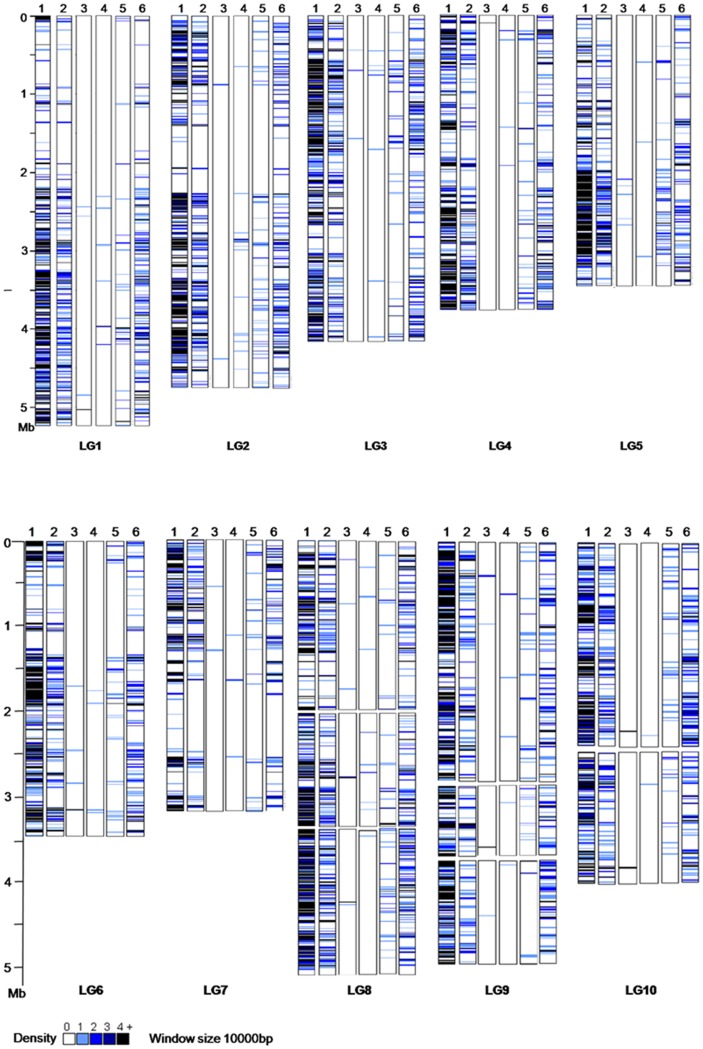
Density of transposable elements (TE) along the linkage groups of *L. bicolor.* The density is graphically represented as the number of TE copy found in a window of 10 kb. Lane 1 represents the density of all TEs; Lane 2 represents the density of *Gypsy* and *Copia*; Lane 3 represents the density of *Helitron*; Lane 4 represents the density of *LARD*; Lane 5 represents the density of LINE; Lane 6 represents the density of TIRs.

Eukaryotic TEs are divided into two classes, depending on their mode of transposition: Class I elements or retroelements or also retrotransposons, which mobilize via a ‘copy-and-paste’ mechanism that uses an ribonucleic acid (RNA) intermediate [12], and class II elements or deoxyribonucleic acid (DNA) transposons, which mobilize via a ‘cut-and-paste’ mechanism that use a DNA intermediate. These two classes are composed of five major types: long terminal repeat (LTR) retrotransposons, non-LTR retrotransposons, cut-and-paste DNA transposons, rolling-circle DNA and self-synthesizing DNA transposons. Each type of TE is composed of a number of superfamilies or clades based on length and target site features, with each superfamily consisting of numerous families. The DNA transposons (class II elements) have terminal inverted repeats (TIRs) or a rolling circle replicon mechanism, e.g., *Helitrons* elements, similar to some known prokaryotic transposition mechanism or self-synthesizing DNA transposons (*Polintons*). The retrotransposons (class I elements) are the most common TE in fungi [13]. As noted above retrotransposons can be classified into two types – LTR retrotransposons and non-LTR retrotransposons (encompassing LINE elements), depending whether they possess or lack long terminal repeats (LTRs) at both ends [13], tyrosine recombinase retroelements (YR; subdivided in three families, *DIRS*, *Ngaro* and *VIPER*), Penelope-like retrotransposable elements, and short interspersed nuclear elements (SINEs) [14]. The LTR retrotransposons, which have a long terminal repeat at their extremities, have been divided into superfamilies: vertebrate retroviruses (Retroviridae), hepadnaviruses, caulimoviruses, *Ty1-Copia-like* (Pseudoviridae), *Ty3-Gypsy-like* (Metaviridae) and *Pao-BEL-like* depending on their sequence similarity and the type of gene products they encode. The two main superfamilies of LTR retrotransposons found in fungi [13] are *Gypsy* and *Copia,* which differ in the order of reverse transcriptase (RT), ribonuclease H (RH), and integrase (IN) domains in the virus-like polyprotein (POL; *Gypsy*: PR-RT-RH-INT, *Copia*: PR-INT-RT-RH).

**Figure 2:**
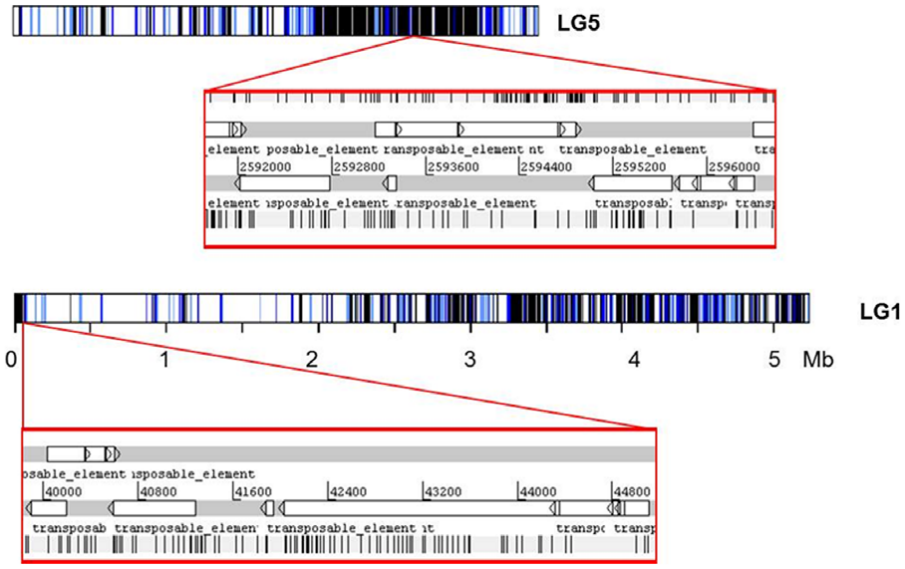
Artemis v11 depiction showing two samples of the nested and clustered distribution of transposable elements (TE) on the *L. bicolor* LG 5 and LG 1.

**Figure 3:**
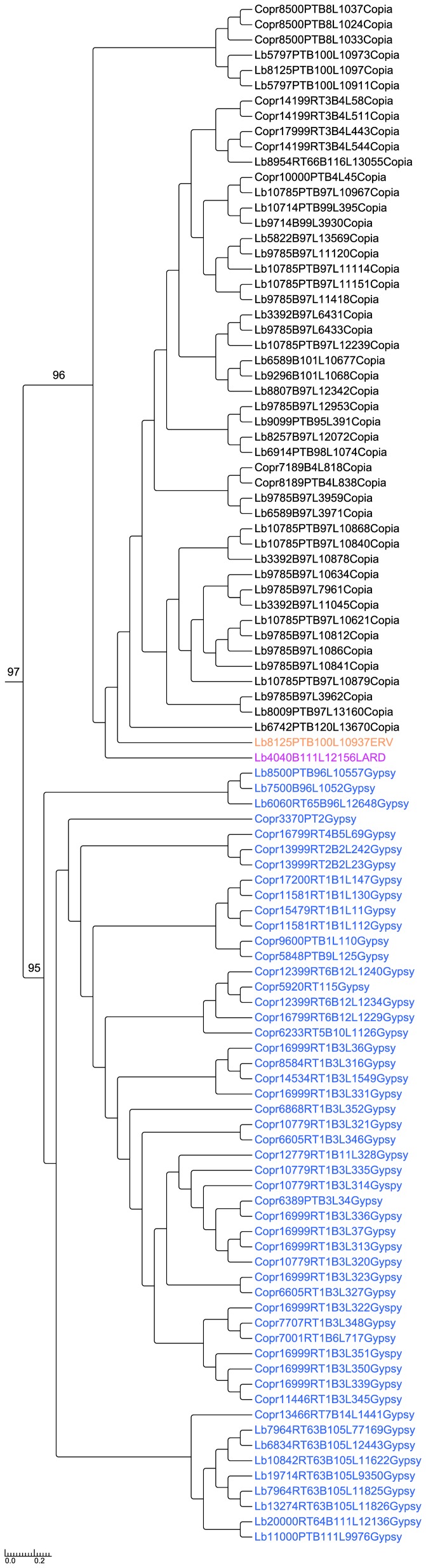
Relationships between LTR retrotransposons of *Laccaria bicolor* and *Coprinopsis cinerea.* This phylogenetic tree is based on reverse transcriptase (RT) and ribonuclease H (RH) amino-acid sequences of all the LTR retrotransposons identified by LTR_STRUC and after manual curation. In black: clade 1, in blue: clade 2. *Gypsy* elements are designated by blue font, *Copia* by black, *LARD* by magenta and *ERV* by orange.

**Figure 4:**
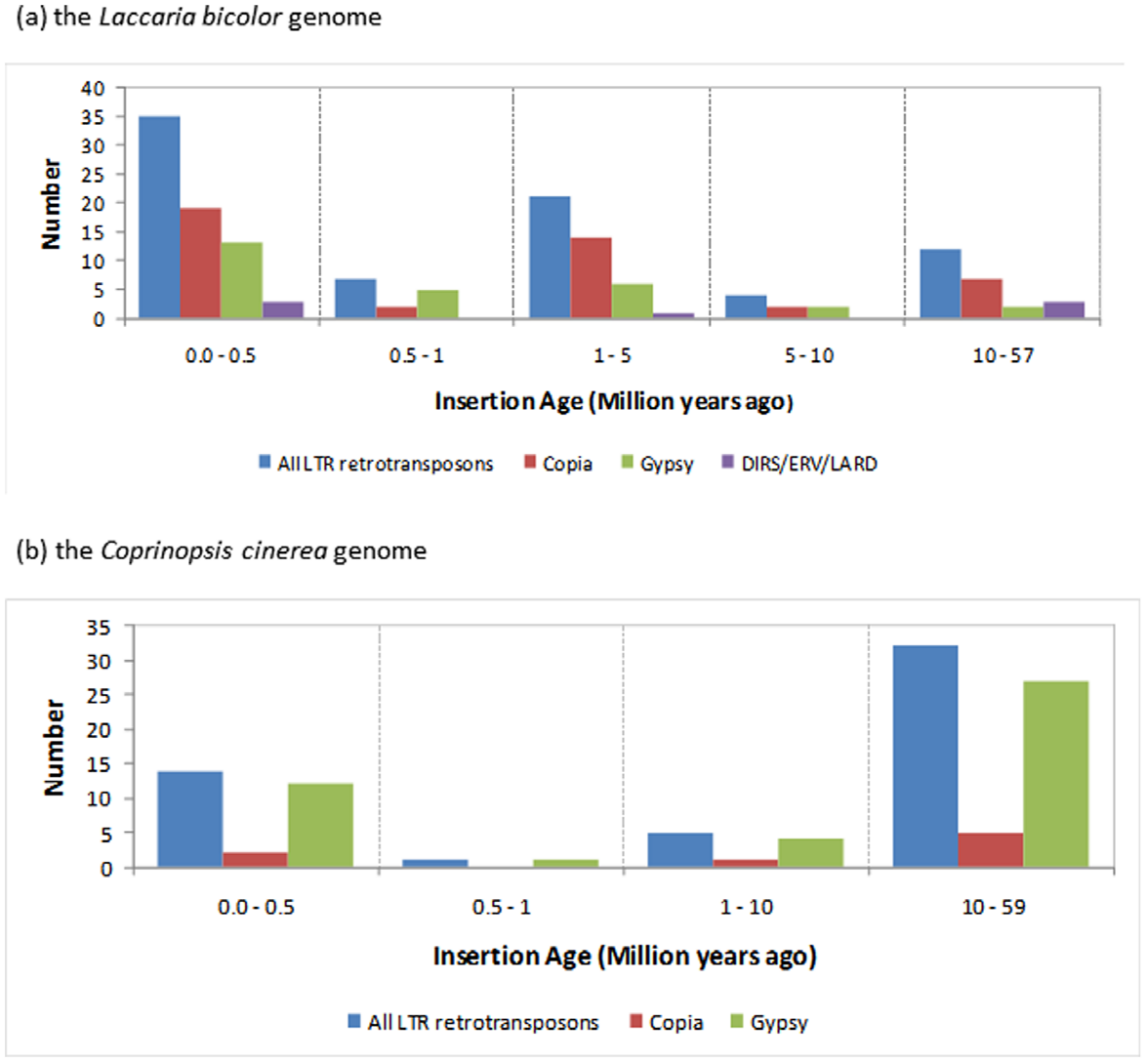
Number of LTR retrotransposon families relative to their insertion age expressed in million years.

Fungi typically have small genomes in the 10 to 40 Mb range, usually with limited amounts of repetitive DNA [15] and thus the Ascomycota and Basidiomycota appear to have a tendency towards streamlined genomes. The majority of these taxa contain no more than 10–15% repetitive DNA [16], [17]. However, the haploid genome of *L. bicolor* comprised a high proportion of repetitive elements relative to other fungal genomes [2].

As TEs are mobile, profuse, variable and dispersed throughout the genome, they can cause serious difficulties in sequence assembly. To assemble and annotate the *L. bicolor* genome it was necessary to determine the abundance and distribution of TEs. Here, we report the automated annotation and manual curation of TEs in *L. bicolor* genome (v2.0). We investigate the diversity and distribution for all TE types and temporal feature for the LTR retrotransposons. In addition, we compare LTR retrotansposons spatial distribution and insertion age of full-length LTR retrotransposons in *L. bicolor*, with the taxonomically related saprophytic Agaricales *Coprinopsis cinerea*.

## Materials and Methods

Sequences analyses were performed using the whole-genome sequence (WGS) version 2 of the homokaryotic (haploid) strain S238N-H82 from *L. bicolor* (Agaricomycetes, Agaricales, Hydnangiaceae) downloaded from the Joint Genome Institute (http://genome.jgi-psf.org/Lacbi2/Lacbi2.download.html). The WGS of *Coprinopsis cinerea* (Agaricomycetes, Agaricales, Psathyrellaceae) was downloaded from the Broad Institute (http://www.broadinstitute.org/). The known TE reference set was constructed from 1) RepeatMasker repeat library (http://www.repeatmasker.org), 2) RepBase on the Genetic Information Research Institute website (GIRI) (http://www.girinst.org/repbase/index.html) and 3) sequences from NCBI (http://www.ncbi.nlm.nih.gov/).

### Sequence annotation

TEs were annotated in the *L. bicolor* genome using the RMBLR procedure from the TE annotation pipeline described by Quesneville *et* *al.* [18]. Consecutive fragments on both the genome and the reference TE were automatically joined if they were separated by a sequence of which more than 80% consisted of other TE insertions. Simple repeats were found using the Tandem Repeat Finder program [19] and used to filter out spurious hits. All TE annotations that were less than 20 bp, after removing any regions that overlapped simple repeat regions, were eliminated. Finally, 171 consensus sequences (complete and incomplete) belonging to various classes or types of TEs were obtained. LTR retrotransposons sequences were confirmed using a second identification procedure based on the program LTR_STRUC [20]. To facilitate manual curation we promoted the 171 consensus sequences identified via RMBLR to a candidate annotation set (defined as a set of one or more joined fragments and by using sequence alignments with CLUSTALW (http://www.ebi.ac.uk/clustalw) that were then validated or modified by the curator in Artemis v11. The resulting database of the annotated TEs of *L. bicolor* is available at http://mycor.nancy.inra.fr/IMGC/LaccariaGenome/download.php?select=anno.

### TE copy number estimation and distribution

The copy number for each TE type was calculated on the basis of the elements obtained from the TE annotation pipeline, TBLASTN searches and RepeatMasker analysis used in the previous pipeline [2]. The TE distribution in *L. bicolor* assembly was determined via RepeatMasker analysis using our curated and annotated *L. bicolor* TE database. Python scripts were used for the mapping of TE density on the assembly anchored on the updated genetic linkage groups (LG) from the genetic map of *L. bicolor* [21]. Multiple sequence alignments were constructed using CLUSTALX [22] to generate nucleotide sequence similarity between LTR sequences of LTR retrotransposon full-length copies. The sequence divergence (in %) was then used to estimate the relative age of TEs.

### Sequence alignments and comparison

To study the phylogenetic relationship between the major elements form in *L. bicolor* and *C. cinerea*, the reverse transcriptase (RT) and the ribonuclease H (RH) amino acid sequences were aligned for all full-length, manually curated LTR elements. Multiple sequence alignments were constructed with CLUSTALX [22]. Phylogenetic trees were constructed on the basis of the neighbor-joining method using PHYLIP with default parameters [23], [24]. Bootstrap values were estimated for each tree from 1000 replicates. The phylogram graphics were created using FigTree v1.2.3 (http://tree.bio.ed.ac.uk/software/figtree/).

The insertion age of full-length LTR retrotransposons was determined by comparing their 5′ and 3′ LTR sequences and based on the assumption that the two LTRs of a single element are identical at the time of the insertion. The two LTRs were aligned with CLUSTALX software [22] and the number of transition and transversion mutation were counted. The insertion times were dated using the Kimura two-parameter method (emboss distmat, http://150.185.138.86/cgi-bin/emboss/distmat). The time (*T*) since element insertion for each family of retroelements (e.g., LTR *Gypsy* family 1 vs. LTR *Gypsy* family 2 found in *L. bicolor*) was estimated using the formula *T*  =  *K2P/2r, where K*  =  evolutionary distance per site, *P*  =  probability of transition *and r*  =  probability of identity at homologous sites [25]. For the conversion of sequence distance to insertion age, an average substitution rate of 1.3E^−8^ mutations per site per year was used based on estimates by Ma and Bennetzen [26] and considered close enough to the molecular clock of LTR retrotransposons in fungi [27].

## Results

### TE abundance, diversity and distribution

Approximately 15 Mb (24%) of the 60 Mb of *L. bicolor* genomic assembly sequence is comprised of TEs (http://mycor.nancy.inra.fr/fr/index.html). Among the 15 Mb, TEs of class I represent 10% of the genome (∼6 Mb) and TEs of class II represent 5% (∼3 Mb). The remaining 5 Mb (9% of the genome) were truncated or did not match classified TEs. Consensus sequences obtained by the TE RMBLR annotation pipeline, used as queries in BLASTN searches of *L. bicolor* genomic sequence, did not contain any additional repeats, indicating that the vast majority of TEs had been identified. The results of these analyses are summarized in Table 1 and presented in detail in Table S1.

From the 171 consensus sequences obtained by the RMBLR procedure, excluding 56 TE fragments (each fragment representing a potential TE family; 11,070 copies), we identified 115 families of TEs in *L. bicolor* containing 930 full-length copies and 13,787 incomplete copies. The major were in the class I (87 families) cluster, in which the *Copia* families were the most numerous (46 members), followed by *Gypsy* (27 members). The third most abundant group was represented by the TIRs (class II) with 23 members. We also identified two *Tad*, one *Deceiver*, one *L1*, five LINEs, four *MITEs*, three large retrotransposon derivatives (*LARD*), one *ERV*, one *DIRS*, and one *Helitron*. The *Helitron*, as well as the *MITEs* and *LARD*, were primarily found in gene-rich regions coding for signal transducers, nucleotide binding proteins, proteins G and RNA binding proteins (data not shown). An intact copy of *LARD* was also located less than 1 kb from the 3′end of the *B* mating type locus following by an intact *MITE* [28].

The identification of thousands of TEs in the *L. bicolor* genome provided the informatics resource to address questions about TE diversity and temporal aspect of TE amplification. We compared all TE types and families previously identified by multiple alignments using CLUSTALX [22]. According to sequence diversity metrics, most of the TE types and families originate in old transposition events except for the intact copies of four TIRs, two *MITEs*, three LTR retrotransposons and one *LARD*, which represent more recent events and in total encompass 5% of the genome (Table S1). These elements contained nearly identical members (threshold >95% identity), suggesting they are potentially still active.

Along the 10 *L. bicolor* pseudochromosomes (designated LG1 to 10), the average density of TEs across the whole-genome was 430 copies per Mb. TE density was not correlated with the chromosome size (r = 0.2), though TE density was higher in the unmapped small scaffolds, likely reflecting the difficulty of unambiguously aligning and assembling contigs with high repeat content. To determine whether TEs might display non-uniform accumulation across the genome, we analyzed their distribution within a sliding 10 kb intervals along the anchored genetic-physical LGs of *L. bicolor* representing 47.7 Mb of the total 60 Mb. Due to their small length, the unmapped scaffolds were not included in this analysis. As shown in Figure 1, TEs were not uniformly distributed within or among LGs. Similar results were obtained within 100 kb intervals (data not shown). Each LG contained regions exhibiting high TE density, mainly *Copia, Gypsy* and TIR elements. In general, these regions corresponded to the ends of the LGs and were found most abundantly in the telomeric regions of LG2, LG4, LG6 and LG9.

By convention [29], [30], TEs inserted within another transposable element are defined as nests, and groups of TEs located within 10 kb of each other are defined as clusters. We found 382 nests and/or clusters of TEs containing 138 families (e.g., Figure 2), suggesting that about 80% of TEs of *L. bicolor* were either inserted into another element and/or were positioned adjacently to another element. Among the 382 nests or clusters, 153 were within the terminal 1 Mb region of the LGs. Most of TEs belonging to nests or clusters were incomplete. LTR retrotransposons *Gypsy* and *Copia* (34%) were nested or clustered more often than either TIRs (25%) or LINE (16%), which is roughly proportional to their relative abundance within the genome. As previously reported [2], this nested structure was predominant in weakly recombinant telomeric regions, where TE insertion may produce rearrangements without deleterious effects.

### Phylogenetic analysis of the LTR retrotransposons

Given that LTR retrotransposons are the most frequent TEs in the *L. bicolor* genome (*Copia* > *Gypsy*), we analyzed the relationships between these elements and those of *C. cinerea* where LTR retrotransposons are also over-represented (*Gypsy* > *Copia*). We constructed phylogenetic trees based on multiple alignments of reverse transcriptase (RT) and ribonuclease H (RH) domains including all identified families of LTR retrotransposons on both genome sequences using LTR_STRUC (Figure 3) [20]. *L. bicolor* and *C. cinerea* TEs generally exhibited the same phylogenetic structure each containing two independent monophyletic clades each supported with bootstrap values >90. In *L. bicolor*, as well as in *C. cinerea*, clade 1 is mainly comprised of *Copia* elements, while clade 2 is comprised of *Gypsy* (Figure 3). The RT and RH sequences of most of *Gypsy* and *Copia* families appeared to be closely related to each other in both genomes (Figure 3). Interestingly, the *ERV* element appears in separate clade, suggesting independent origins of this TE (Figure 3).

### The insertion age of LTR retrotransposons

As shown by Figure 4A and 4B, the oldest detected LTR retrotransposon insertion occurred in the genome of the ancestor of *L. bicolor* and *C. cinerea* 57 and 59 million years ago (Mya). In *L. bicolor*, there were three main periods of expansion of TE families: the first between 57 and 10 Mya, the second between 5 and 1 Mya and the last from 500,000 years ago until present. In *C. cinerea*, there were only two main periods of TE expansion: the first between 59 and 10 Mya and a second from 0.5 Mya ago until now. In *L. bicolor*, during the first two periods *Copia* expansion was more abundant than *Gypsy*, but between 0.5 to 1 Mya *Gypsy* expansion was more abundant than *Copia*. In contrast, in *C. cinerea* during the two main periods of TE expansion, *Gypsy* expansion was consistently more abundant than that for *Copia*.

## Discussion

The *L. bicolor* genome is rich in TEs (24% of the genome), which partially contributes to its relative large genome size compared to other Basidiomycota. Indeed a clear connection is emerging from genome comparisons: the larger the genome, the higher the number of TEs [31]. Analogous considerations can be extended from the 60 Mb of *L. bicolor* to the 140 Mb of the Ascomycota *Tuber melanosporum* (58% of TEs) [32].

The largest class of TEs highlighted in this study was the class I elements, with a high proportion of LTR retrotransposons (*Copia* > *Gypsy*), which confirms Muszewska *et* *al.* observations [33]. Moreover, we identified additional TE types such as *LINE*, *ERV* and *LARD* and we established the presence of *Tad*, *Deceiver* and *L1* non-LTR elements previously suggested in *L. bicolor* by Novikova *et* *al*. [34]. A large proportion of class II elements also were found, mainly TIRs, *Helitron* and *MITEs*. In *L. bicolor* the single *Helitron* and its related copies account for about 0.13% of the genome and are mainly found in comparatively gene-rich regions, as has been reported in maize [31], while conversely they appear to be preferentially localized in the gene-poor region in *Arabidopsis* and rice genomes [35], [36]. In either case, the characteristics of *Helitrons* make them one of the primary sources of DNA variation [35]. Despite the small size and low proportion of *Helitrons* in the *L. bicolor* genome, its presence in gene-rich regions suggests this element may be involved in introducing sequence variation within coding regions in this genome.

*MITEs*, among the smallest TEs, and *LARDs* have been more commonly associated with genes than are other types of TEs in plants [37], [38], [39], [40]. In *L. bicolor MITE*s and *LARD*s represent 0.10% and 0.08% of the genome, respectively, and were found among gene-rich regions. This gene-TE association may have implications for gene regulation, mRNA stability or conservation of expression of loci in large multigenic families [12], and as has been related to R genes in rice [41]. The occurrence of intact, i.e., recent, *LARD* and *MITE* in the 3′end of the *L. bicolor B-*mating type locus suggests a possible role of this element in the rapid evolution of the *B*-mating type containing region. Interestingly, Niculita-Hirzel *et* *al.* [28] observed a similar distribution of the transposon insertion around the *B*-containing region and not around the *A*-mating type.

Among the 171 TE families, 43 individual elements exhibited intact structure, suggesting that these elements are still active. This hypothesis is supported by expression evidence corresponding to their transcripts detected in free-living mycelium, fruiting bodies and ectomycorrhizas [2]. However, it has been proposed that fungal cells may control transposition at a post-transcriptional level.

Phylogenetic analysis of the LTR retrotransposons revealed that these elements are closely related amongst themselves and to retrotransposons found in another Agaricales, i.e., *C. cinerea*, as Muszewska *et* *al.* [33] has shown. These elements might descend from a common ancestor of *L. bicolor* and *C. cinerea* that split from their common ancestor ∼90 Mya ago (D Hibbett, personal communication). Our estimation of the insertion age of LTR retrotransposons suggests that *L. bicolor* ancestors experienced three main bursts of LTR retrotransposon expansion, including a recent one. *C. cinerea* ancestor had only two main periods of expansion, which could explain the higher frequency of LTRs observed in *L. bicolor*. Estimation of insertion age, as well as phylogenetic tree, revealed a recent expansion of *Copia* elements in *L*. *bicolor*, while the *Gypsy* elements expansion appeared to be more ancient. The first main period of TE in *L. bicolor* ancestor dates from 57 Mya. According to Ryberg and Matheny [42], Hydnangiaceae appeared before 40 Mya, much before *L. bicolor* evolved. The *L. bicolor* and *L. nobilis* species appears to have evolved from *L. laccata* 12–14 Mya [42], [43], supporting our contention that the second LTR retrotransposon expansion might have coincided with the species divergence.

TEs are not randomly distributed across the *L. bicolor* genome, but are tightly nested or clustered. The analysis of the TE spatial distribution on the LGs revealed that the nests and/or clusters are mostly positioned at the proximal ends in the telomeric regions. The majority of TEs belonging to nests or clusters are mainly LTR retrotransposons (*Copia* and *Gypsy*), and to a lesser degree, *TIR*s and *LINE*. There are several mechanisms that may explain this type of distribution. The ectopic exchange model states that repetitive DNA promotes deleterious ectopic recombination events [44]. Since selection pressure would be reduced in regions of low recombination, repetitive DNA should accumulate in such regions. Under the insertion model, TE insertions generally have a deleterious effect on fitness and are thus likely to be lost from the population [12]. That is, in chromosomal segments that have low recombination rates, deleterious mutations are more likely to be genetically linked to neutral genes, decreasing their negative fitness effect. The insertion of TE may trigger a runaway process, where there would be a cascade displacement of TEs, as each TE would provide a target into which other elements could be insert without deleterious consequences [45].

The insertion model is supported by the observation that TE occurrence was highest in regions of the genome with high microsatellite density [46]. It has been suggested that TE-associated microsatellites (SSRs) are either components of active TEs spreading throughout the genome, acting as a ‘landing pad’ for TE insertion or they arise after the integration of an extended and polyadenylated retro-transcript into the genome [47], [48]. Arcot et al. [49], Ellegren [50] and Zhu et al. [51] have shown that *LINE*s, *LTR*s and *MITEs* were able to generate and propagate microsatellites. Thus, the distribution of TEs in clusters could result from potential insertion sites formed by microsatellites flanking these TEs that may subsequently lead to accumulation of chromosome deletions through unequal homologous recombination or interchromosomal recombination [52], [53].

As in all plant, animal and fungal genomes characterized to date, the *L. bicolor* and *C. cinerea* genomes have a distinctive TE composition and comparative evolutionary history. The high proportion, the large diversity, and the nested and clustered distribution of TE in *L. bicolor* suggest rearrangements promoted by their occurrence. This nested and clustered distribution is associated with SSR occurrence and could be the result of potential insertion sites formed by the SSRs flanking the TEs [54], [47]. The great diversity of TEs in *L. bicolor* genome can be partially explained by the several periods of expansion of TEs but may also be the result of potential horizontal transfer. Horizontal transfer is the exchange of genetic information between reproductively isolated organisms and has been shown to be of significance in the evolution of some functional fungal genes [55], [56], [57]. Diversity of TEs and their copy number depends on the evolutionary history of a particular species or a cluster of closely related species, their population structure and ecological features. There are several main processes which could affect the copy number and diversity of TEs in fungal genomes: stochastic loss of elements, burst of transposition, the limitation of copy number increase by natural selection which removes deleterious insertions, passive and active inactivation of repetitive sequences, and self-regulation of transposition (decrease of the transposition rate when the copy number increases) [58]. The population structure and dynamics, as well as mating mode, also play an important role in the transposable elements evolution. The inactivation of repeated sequences is also a very important factor, which leads to the shifts in diversity and copy number of TEs, especially in fungi. Sequencing of additional strains of *L. bicolor* and *Laccaria* species should provide the needed information to investigate the mechanisms in action in *Laccaria*.

TEs are distributed throughout the *L. bicolor* genome and could be major contributors to the genesis of new genes or to the adaptation of existing genes, notably via mechanisms such as molecular domestication, ectopic recombination and gene retrotransposition. Molecular domestication, also known as the process of TE recruitment by the host genome, is the coopted use by the organism of a function carried by a TE. Because TEs encode proteins that can for example bind, copy, break, join or degrade nucleic acids, they have been repeatedly domesticated during eukaryotic evolution [59]. As another mechanism, retrotransposon-mediated ectopic recombination results from the physical occurrence of retrotransposon insertions at particular sites in the genome and can imply various genomic rearrangements, such as duplications, deletions and translocations. Gene retrotransposition is also another mechanism that can rearrange genes. Gene retrotransposition operates during the retrotransposition process itself and only duplicates gene sequences but no retrotransposon sequence. Thus, movement of TEs increasing their density in the *L. bicolor* genome would support the theory that the higher than normal expansion of certain gene families in this genome. Notably, this may have created paralogous genes such as hydrophobin-coding-genes [60], important in host-fungal interactions and giving then a selective advantage to this mutualistic fungus in colonizing a wide range of host plants.

Finally, the high density of TEs in *L. bicolor* genome could be associated with its host plasticity and its evolution from a saprophytic to a symbiotic habit or with TE proliferation after the split between the saprotrophic ancestor and ectomycorrhizal ancestor of *Laccaria*. Each of the above molecular mechanisms appears to have driven the genome expansion in *L*. *bicolor*, conferring increased genome reorganization and possibly leading to genome divergence among related strains. Genome plasticity and high variability among strains suggest that a single reference genome for each species will be insufficient to completely describe and understand the genetic complement of a particular species [54]. Characterizing interspecific differences in TEs among various *Laccaria* species and isolates will be aided by the present study. A *Laccaria* Pan-Genome project is underway which will sequence the genomes of 12 *L. bicolor* strains from geographically diverse origins as well as four related *Laccaria* species. The assignment of TE activity (*Helitron*s insertions, and recombination events) inside the intergenic and intronic regions in these samples will clarify the role of TEs in the evolution and genome organization of ectomycorrhizal genus. The resource developed here will contribute to the extraction of information and/or confirmation of hypotheses concerning genome reorganization and adaptive mutations, as well as act as a reference for shared sequences and differences among genomes within and among related strains.

## Supporting Information

Table S1 **Table showing the percentage of identity of multiple alignment of TE copies corresponding to 175 consensus TE families identified by the RMBLR procedure.**
(DOCX)
