# Supplementary material for: Characterization of Transposable Elements in the Ectomycorrhizal Fungus Laccaria bicolor
Source: PLoS One. 2012 Aug 3;7(8):e40197. doi: 10.1371/journal.pone.0040197 (PMC3411680; doi:10.1371/journal.pone.0040197)
Supplement: Table S1 — Table showing the percentage of identity of multiple alignment of TE copies corresponding to 175 consensus TE families identified by the RMBLR procedure. (DOCX) [file pone.0040197.s001.docx]

**Table S1.** **Table showing the percentage of identity of multiple alignment of TE copies corresponding to 175 consensus TE families identified by the RMBLR procedure.**

| **TE family ID** | **Class** | **Type** | **Pairwise multiple alignment identity (%)** |
| --- | --- | --- | --- |
| ReconFam1562 | II | TIR | 98 |
| T_scf9_3 | I | LTR | 97 |
| ReconFam30 | II | TIR | 97 |
| ReconFam33 | II | TIR | 96 |
| ReconFam40 | II | TIR | 96 |
| ReconFam654 | I | LTR | 96 |
| T_scf60_1 | I | LTR | 96 |
| MITE_X2 | II | MITE | 95 |
| MITE_X3 | II | MITE | 95 |
| ReconFam383 | I | LARD | 95 |
| ReconFam1037 | II | TIR | 95 |
| T_scf20_2 | I | LTR | 94 |
| ReconFam43 | II | TIR | 94 |
| ReconFam938 | II | TIR | 94 |
| T_scf49_1 | I | LTR | 93 |
| ReconFam437 | II | TIR | 93 |
| T_scf6_3 | I | LTR | 93 |
| ReconFam243 | II | TIR | 92 |
| HMMReconFam82 | I | Non-LTR | 92 |
| ReconFam641 | II | TIR | 92 |
| ReconFam399 | I | Non-LTR | 91 |
| HMMReconFam1523 | I | Non-LTR | 91 |
| HMMReconFam986 | I | Non-LTR | 91 |
| ReconFam374 | II | TIR | 90 |
| ReconFam1184 | II | TIR | 90 |
| HMMReconFam219 | I | Non-LTR | 90 |
| ReconFam1670 | II | TIR | 90 |
| T_scf4_5 | I | LTR | 90 |
| HMMReconFam622 | I | Non-LTR | 90 |
| ReconFam877 | II | TIR | 89 |

| **TE ID** | **Class** | **Type** | **Pairwise multiple alignment identity (%)** |
| --- | --- | --- | --- |
| ReconFam2358 | II | TIR | 88 |
| T_scf21_1 | I | LTR | 88 |
| T_scf7_1 | I | LTR | 88 |
| ReconFam2355 | II | TIR | 88 |
| ReconFam416 | I | LTR | 88 |
| HMMReconFam572 | no cat | no cat | 87 |
| T_scf52_1 | I | LTR | 87 |
| HMMReconFam3152 | no cat | no cat | 87 |
| ReconFam116 | no cat | no cat | 87 |
| HMMReconFam1417 | no cat | no cat | 86 |
| ReconFam2610 | II | TIR | 86 |
| HMMReconFam228 | no cat | no cat | 86 |
| HMMReconFam611 | no cat | no cat | 86 |
| ReconFam1841 | II | TIR | 85 |
| HMMReconFam691 | no cat | no cat | 85 |
| HMMReconFam315 | no cat | no cat | 85 |
| HMMReconFam395 | no cat | no cat | 85 |
| ReconFam346 | II | TIR | 85 |
| ReconFam924 | II | TIR | 85 |
| ReconFam17 | II | TIR | 85 |
| ReconFam125 | II | TIR | 85 |
| T_scf3_2 | I | LTR | 85 |
| ReconFam3527 | I | LTR | 85 |
| ReconFam559 | no cat | no cat | 85 |
| T_scf91_1 | I | LTR | 84 |
| ReconFam2694 | II | TIR | 84 |
| ReconFam807 | II | TIR | 84 |
| T_scf1_6 | I | LTR | 84 |
| HMMReconFam311 | no cat | no cat | 84 |
| ReconFam18213 | II | MITE | 84 |
| T_scf6_2 | I | LTR | 83 |
| T_scf42_1 | I | LTR | 83 |
| ReconFam316 | I | LTR | 83 |
| HMMReconFam973 | no cat | no cat | 83 |
| HMMReconFam782 | no cat | no cat | 83 |
| HMMReconFam343 | no cat | no cat | 83 |
| T_scf78_1 | I | LTR | 83 |
| ReconFam285 | II | TIR | 83 |
| ReconFam2359 | II | TIR | 83 |

| **TE ID** | **Class** | **Type** | **Pairwise multiple alignment identity (%)** |
| --- | --- | --- | --- |
| ReconFam629 | II | TIR | 83 |
| HMMReconFam1041 | no cat | no cat | 83 |
| ReconFam547 | no cat | no cat | 83 |
| T_scf6_1 | I | LTR | 83 |
| HMMReconFam2191 | I | LARD | 82 |
| T_scf43_1 | I | LTR | 82 |
| HMMReconFam1286 | no cat | no cat | 82 |
| ReconFam838 | II | TIR | 82 |
| T_scf144_1 | I | LTR | 82 |
| ReconFam1049 | II | TIR | 82 |
| MITE_X1 | II | TIR | 82 |
| ReconFam223 | no cat | no cat | 82 |
| HMMReconFam354 | no cat | no cat | 82 |
| HMMReconFam296 | no cat | no cat | 82 |
| T_scf90_1 | I | LTR | 81 |
| T_scf80_2 | I | LTR | 81 |
| HMMReconFam355 | no cat | no cat | 81 |
| HMMReconFam24 | no cat | no cat | 81 |
| HMMReconFam610 | no cat | no cat | 81 |
| ReconFam1019 | I | LTR | 81 |
| T_scf4_4 | I | LTR | 81 |
| T_scf8_1 | I | LTR | 81 |
| HMMReconFam353 | no cat | no cat | 81 |
| ReconFam1142 | no cat | no cat | 81 |
| ReconFam24640 | II | MITE | 81 |
| ReconFam28 | II | TIR | 81 |
| T_scf80_1 | I | LTR | 81 |
| T_scf1_5 | I | LTR | 81 |
| HMMReconFam1053 | no cat | no cat | 81 |
| T_scf8_2 | I | LTR | 80 |
| HMMReconFam229 | no cat | no cat | 80 |
| T_scf23_1 | I | LTR | 80 |
| ReconFam190 | II | TIR | 80 |
| ReconFam2178 | no cat | no cat | 80 |
| MITE_LaTEX1 | II | MITE | 80 |
| ReconFam737 | no cat | no cat | 80 |
| Restless-like | II | TIR | 80 |
| T_scf2_3 | I | LTR | 80 |
| TIR-ET1 | II | TIR | 80 |
| ReconFam158 | II | TIR | 80 |
| ReconFam138 | II | TIR | 80 |

| **TE ID** | **Class** | **Type** | **Pairwise multiple alignment identity (%)** |
| --- | --- | --- | --- |
| HMMReconFam1555 | no cat | no cat | 80 |
| T_scf10_2 | I | LTR | 80 |
| T_scf41_1 | I | LTR | 80 |
| restless? | II | TIR | 80 |
| HMMReconFam1465 | no cat | no cat | 80 |
| T_scf41_2 | I | LTR | 80 |
| ReconFam16 | II | TIR | 79 |
| T_scf14_1 | I | LTR | 79 |
| T_scf1_3 | I | LTR | 79 |
| MITE_X4 | II | MITE | 79 |
| ReconFam730 | II | TIR | 79 |
| T_scf21_3 | I | LTR | 79 |
| ReconFam447 | no cat | no cat | 79 |
| HMMReconFam445 | no cat | no cat | 79 |
| ReconFam301 | II | TIR | 79 |
| HMMReconFam1527 | no cat | no cat | 79 |
| T_scf3_1 | I | LTR | 79 |
| T_scf4_1 | I | LTR | 79 |
| ReconFam244 | no cat | no cat | 79 |
| ReconFam10701 | II | MITE | 79 |
| ReconFam2208 | I | LTR | 79 |
| T_scf21_2 | no cat | no cat | 79 |
| ReconFam217 | I | LTR | 79 |
| ReconFam1180 | II | TIR | 78 |
| ReconFam281 | II | TIR | 78 |
| ReconFam1195 | I | LTR | 78 |
| T_scf18_1 | I | LTR | 78 |
| ReconFam520 | II | TIR | 78 |
| ReconFam391 | II | TIR | 78 |
| HMMReconFam2190 | no cat | no cat | 78 |
| T_scf71_1 | I | LTR | 78 |
| ReconFam419 | II | TIR | 78 |
| ReconFam280 | I | LTR | 78 |
| T_scf74_1 | I | LTR | 78 |
| HMMReconFam233 | no cat | no cat | 78 |
| T_scf38_1 | I | LTR | 78 |
| T_scf40_1 | I | LTR | 78 |
| HMMReconFam693 | I | LINE | 78 |
| ReconFam1269 | I | LTR | 78 |
| T_scf1_1 | I | LTR | 78 |
| T_scf1_4 | I | LTR | 78 |

| **TE ID** | **Class** | **Type** | **Pairwise multiple alignment identity (%)** |
| --- | --- | --- | --- |
| ReconFam1725 | II | TIR | 78 |
| ReconFam702 | I | LINE | 77 |
| HMMReconFam386 | I | LINE | 77 |
| ReconFam1305 | II | Helitron | 77 |
| Fot1-like | II | TIR | 77 |
| HMMReconFam4263 | no cat | no cat | 77 |
| T_scf63_1 | I | LTR | 77 |
| T_scf11_2 | I | LTR | 77 |
| ReconFam510 | no cat | no cat | 76 |
| ReconFam928 | I | LINE | 76 |
| HMMReconFam2229 | no cat | no cat | 76 |
| T_scf24_1 | I | LTR | 75 |
| HMMReconFam1457 | no cat | no cat | 75 |
| T_scf1_2 | I | LTR | 75 |
| HMMReconFam1504 | no cat | no cat | 75 |
| MITE_LaTEX2_master | II | MITE | 75 |
| T_scf47_1 | I | LTR | 75 |
| ReconFam350 | I | LINE | 75 |
| T_scf24_2 | no cat | no cat | 75 |
| HMMReconFam1079 | no cat | no cat | 75 |
| T_scf17_3 | I | LTR | 75 |
| HMMReconFam1300 | no cat | no cat | 74 |
| ReconFam2212 | no cat | no cat | 74 |
| ReconFam726 | II | TIR | 74 |
| T_scf10_1 | I | LTR | 74 |
| TIR-ET3 | II | TIR | 73 |
| T_scf43_2 | I | LTR | 73 |
| tc1-like | II | TIR | 73 |
| HMMReconFam472 | no cat | no cat | 73 |
| HMMReconFam487 | I | LTR | 73 |
| ReconFam1284 | no cat | no cat | 73 |
| HMMReconFam786 | no cat | no cat | 73 |
| ReconFam567 | no cat | no cat | 72 |
| T_scf4_3 | I | LTR | 72 |
| T_scf41_3 | I | LTR | 72 |
| T_scf11_1 | I | LTR | 72 |
| ReconFam1186 | no cat | no cat | 72 |
| HMMReconFam1452 | I | LINE | 71 |
| Nht1-like | II | TIR | 71 |
| T_scf9_1 | I | LTR | 71 |

| **TE ID** | **Class** | **Type** | **Pairwise multiple alignment identity (%)** |
| --- | --- | --- | --- |
| T_scf27_1 | I | LTR | 70 |
| ReconFam1181 | no cat | no cat | 69 |
| HMMReconFam3186 | I | LTR | 69 |
| ReconFam349 | no cat | no cat | 69 |
| T_scf9_2 | I | LTR | 68 |
| ReconFam317 | I | Non-LTR | 68 |
| ReconFam1227 | I | Non-LTR | 68 |
| T_scf37_1 | I | LTR | 31 |
| T_scf15_2 | I | LTR | 29 |
| T_scf2_2 | I | LTR | 28 |
| T_scf61_1 | I | LTR | 28 |
| T_scf20_1 | I | LTR | 26 |
